# Dry Eye Disease Symptoms Among Glaucoma Patients at a Portuguese Hospital

**DOI:** 10.7759/cureus.58249

**Published:** 2024-04-14

**Authors:** Catarina Pestana Aguiar, Pedro Teixeira, Inês Almeida, João Chibante-Pedro, Jeniffer Jesus

**Affiliations:** 1 Ophthalmology Department, Unidade Local de Saúde Entre Douro e Vouga, Santa Maria da Feira, PRT

**Keywords:** glaucoma therapy, dry eye symptoms, eye drops, open angle glaucoma, dry eye disease (ded)

## Abstract

Introduction: Glaucoma-related dry eye disease (DED) is often underestimated, but it is an important comorbidity affecting 40% to 59% of glaucoma patients. It may be an exacerbation of a pre-existing condition or a novel disease starting after the initiation of topical medication. The cumulative effect of medication, preservatives and excipients leads to an alteration in tear film composition and ocular surface stability. The main purpose of this investigation was to study a group of Portuguese glaucoma patients regarding the presence of DED symptoms and correlate the severity of the symptoms with the usage of different types of glaucoma topical medications.

Materials and methods: This is a cross-sectional observational study of patients diagnosed with primary and secondary open-angle glaucoma. The questionnaire Standardized Patient Evaluation of Eye Dryness (SPEED) translated to Portuguese (SPEED-Vp) was taken by patients followed in the Glaucoma Department of Unidade Local de Saúde Entre Douro e Vouga, Santa Maria da Feira, Portugal. Data was collected regarding their age, gender, type of topical medication in use as well as frequency and duration of usage. A statistical analysis was performed.

Results: A total of 75 patients answered the SPEED-Vp questionnaire. The mean age was 72 ± 7 years old. Fifty-two percent (n=39) were male, and 48% (n=36) were female patients. About 49.33% (n=37) had been on intraocular pressure (IOP)-lowering eyedrops for more than five years. About 61.43% (n=43) of patients used IOP-lowering eyedrops with preservatives. Most of the patients used prostaglandin analogs (75.71%, n=53) and beta-blockers (72.86%, n=51). SPEED score average was 2.75. About 25.33% (n=19) had no DED symptoms, 58.67% (n=44) had mild symptoms, 8% (n=6) had moderate symptoms and 8% (n=6) had severe symptoms. No statistically significant correlation was found between SPEED score and age, gender, number of eyedrop containers, number of active principles, application frequency, presence of preservatives, number of eyedrop containers with preservatives, duration of eyedrops usage or any of the medication groups.

Conclusion: Although a high percentage of patients were on eyedrops with preservatives, this low rate of symptoms might be because patients tended to devalue these symptoms; were already on treatment with artificial tears; or have an underestimation of the sensation of dry eye due to decreased neuronal corneal nerve responses and density. These results were surprisingly positive. This might also be the result of the healthcare provider’s sensibilization to this issue (early diagnosis, early prescription of artificial tears and change from preservative to preservative-free medication).

## Introduction

Dry eye disease (DED) is a multifactorial ocular surface disease in which hyperosmolarity and tear film instability play a role [1-3]. Glaucoma-related DED is often underestimated, but it is an important comorbidity affecting 40% to 59% of glaucoma patients worldwide [4] and can have a significant impact on the quality of life [3]. Studies show that DED occurs at a higher rate in patients with glaucoma [3,5-7] and that it is more prevalent in older ages and women [3]. DED in glaucoma patients may be an exacerbation of a pre-existing condition or a novel disease starting after the initiation of topical medication, only within three months of therapy [2]. The cumulative effect of medication, preservatives and excipients leads to an alteration in tear film composition and ocular surface stability (inflammation and increased tear evaporation from meibomian gland dysfunction). In return, these chronic inflammatory changes decrease the efficacy of glaucoma treatment, patient compliance and quality of life [4].

Glaucoma may be associated with DED even without the usage of topical medication, with studies suggesting a lower basal tear turnover in eyes with untreated primary open-angle glaucoma than that of eyes without glaucoma [3]. The usage of multiple medications, the number of drops instilled per day and the duration of therapy were found to have a positive correlation with DED signs, with continuous increasing of the prevalence and severity. Preservatives present in eyedrops have also been associated with the presence of DED; however, it has been shown that preservative-free medication may also lead to this ocular surface disease, which means that either the added preservative or the active ingredient itself can cause or worsen DED [2,3]. The main purpose of this investigation was to study a group of Portuguese glaucoma patients regarding the presence of DED symptoms and correlate the severity of the symptoms with the usage of different types of glaucoma topical medications.

## Materials and methods

We performed a cross-sectional observational study between June and July 2023. We included patients diagnosed with primary and secondary open-angle glaucoma followed by the Glaucoma Department of Unidade Local de Saúde Entre Douro e Vouga, Santa Maria da Feira, Portugal. Patients to whom the questionnaire was given were selected from the list of patients with this diagnosis through systematic sampling. We included 75 patients in the study.

We conducted the questionnaire Standardized Patient Evaluation of Eye Dryness (SPEED) translated to Portuguese (SPEED-Vp) [8] to the above-mentioned patients. The questionnaire was taken through telephone, and patients were asked direct questions and were given direct hypothesis of answers. The questionnaire evaluates not only patients’ current symptoms related to eye dryness but also in the previous months. It evaluates the frequency and severity of four typical symptoms of dry eye disease, namely “dryness, grittiness or scratchiness”, “soreness or irritation”, “burning or watering” and “eye fatigue”, by asking whether they felt these symptoms today, in the previous 72 hours or in the previous three months. For each symptom, the patient classified it regarding its frequency, indicated if they “never” felt it (zero points), if they felt it “sometimes” (one point), if they felt it “often” (two points) or “constant” (three points); and regarding its severity “no problems” (zero points), “tolerable” (one point), “uncomfortable” (two points), “bothersome” (three points) and “intolerable” (four points). After taking the questionnaires, we added up the scores. We considered the following classification: no DED, a score of 0; mild DED, a score of 1-4; moderate DED, a score of 5-7; and severe DED, a score of 8 or more.

At the same time the questionnaire was taken, data was collected regarding patients’ detailed medical history and demographic data, which included age, gender, type of topical medication (eyedrops containing prostaglandin analogs, beta-blockers, carbonic anhydrase inhibitors or alpha-adrenergic agonists, alone or in associations between them) as well as frequency of application (once, twice or three times per day) and duration of usage (less than one year, from two to five years and more than five years). Data regarding previous ocular surgery, namely glaucoma surgery, was also obtained. Patients were also asked whether they knew about the eyedrops side effects and if they could enumerate them.

A statistical analysis was done using the program Jeffreys's Amazing Statistics Program (JASP) 18.3 (Amsterdam, The Netherlands). We correlated the continuous variables with the SPEED score through Pearson’s correlation test or through Spearman’s correlation test when the variables did not respect normality. We correlated the categorical variables with the SPEED score through Spearman’s correlation test. Continuous variables are presented as mean, and categorical variables are presented as percentage. A p-value inferior to 0.05 was considered statistically significant in all tests executed. Most of the statistical analysis is presented in tables to provide better readability. Previously to the questionnaire being taken, all patients have given their informed consent to participate in the study, and the research adhered to the tenets of the Declaration of Helsinki.

## Results

A total of 75 patients answered the SPEED-Vp questionnaire. The mean age was 72 ± 7 years old. Fifty-two percent (n=39) were male, and 48% (n=36) were female patients. From the total of patients inquired, five (0.07%) had already had surgery for intraocular pressure (IOP) control so they were no longer on topical treatment with IOP-lowering medication.

Regarding treatment duration, 49.33% (n=37) had been on IOP-lowering eyedrops for more than five years, 41.33% (n=31) from two to five years and 9.33% (n=7) for less than one year. The number of eyedrop containers, number of active principles and application frequency are present in Table 1.

**Table 1:** Description of IOP-lowering medication used by the sample. IOP: intraocular pressure.

Variable		% (n)
Number of eyedrop containers (n=75)	Zero	6.67 (5)
	One	42.67 (32)
	Two	44.00 (33)
	Three	6.67 (5)
Number of active principles (n=75)	Zero	6.67 (5)
	One	22.86 (16)
	Two	28.57 (20)
	Three	30.00 (21)
	Four	18.57 (13)
Application frequency (n=75)	None	6.67 (5)
	Once a day	22.67 (17)
	Twice a day	70.67 (53)

Regarding the presence of preservatives, 61.43% (n=43) of patients used IOP-lowering eyedrops with preservatives. From these, 69.77% (n=30) used one eyedrop container with preservatives and the remaining 30.23% (n=13) used two eyedrop containers. Most of the patients used prostaglandin analogs (75.71%, n=53) and beta-blockers (72.86%, n=51). Carbonic anhydrase inhibitors were used by 60% (n=42) followed by alpha-adrenergic agonists (35.71%, n=25). When asked, 87.14% (n=61) did not know the adverse effects of the eyedrops they were on. SPEED questionnaire answers are categorized in Table 2.

**Table 2:** SPEED questionnaire answers. SPEED: Standardized Patient Evaluation of Eye Dryness.

Symptom	% (n)	Symptom frequency	% (n)
Dryness, Grittiness or Scratchiness	36 (27)	At this visit	5.33 (4)
		Within the past 72 hours	8 (6)
		Within the past 3 months	22.67 (17)
Soreness or Irritation	18.67 (14)	At this visit	1.33 (1)
		Within the past 72 hours	2.67 (2)
		Within the past 3 months	14.67 (11)
Burning or Watering	40 (30)	At this visit	9.33 (7)
		Within the past 72 hours	16 (12)
		Within the past 3 months	14.67 (11)
Eye Fatigue	5.33 (4)	At this visit	0 (0)
		Within the past 72 hours	4 (3)
		Within the past 3 months	1.33 (1)

SPEED score average was 2.75. About 25.33% (n=19) had no DED symptoms, 58.67% (n=44) had mild symptoms, 8% (n=6) had moderate symptoms and 8% (n=6) had severe symptoms. When performing the correlations between the different variables and the scores, the results are presented in Table 3.

**Table 3:** Correlations between SPEED score and the different variables evaluated. All the analyses were performed using Spearman’s correlation test. SPEED: Standardized Patient Evaluation of Eye Dryness.

Variable	SPEED Score	p-value
Age	-0.048	0.683
Gender	0.103	0.381
Number of eyedrop containers	0.110	0.347
Number of active principles	0.146	0.229
Application frequency	0.175	0.133
Presence of preservatives	0.196	0.103
Number of eyedrop containers with preservatives	0.196	0.105
Duration of eyedrops usage	-0.168	0.158
Prostaglandin analogs	-0.021	0.862
Beta-blockers	0.032	0.793
Alpha-adrenergic agonists	0.221	0.066
Carbonic anhydrase inhibitors	0.085	0.486

## Discussion

Studies show a relationship between the presence of preservatives, the number and prolonged use of glaucoma medications and the presence of ocular surface disease [3]. However, in our investigation, we could not find any statistically significant correlation between the variables studied and the SPEED questionnaire results.

Benzalkonium chloride (BAK) is one of the most commonly used preservatives in IOP-lowering medications because it has antimicrobial properties through its action as a detergent that disrupts bacterial cell walls and prevents the growth of pathogenic bacteria in eyedrop containers [3,4,9]. However, it is associated with a disruption of the corneal surface with corneal epithelial punctate erosion and shorter tear breakup time and may also cause alterations in the microorganisms of the ocular surface which can in turn lead to inflammation [3,9,10]. Other preservatives such as Polyquad, Purite, and sofZia seem to have less toxic effects [2,11]. In our study, we could not find a statistically significant relationship between a higher SPEED score and the usage of eyedrop containers with preservatives, even though we observed that most patients (61.43%) used this type of medication.

Even in the absence of preservatives, some studies show that glaucoma medication is associated with DED [3,12-14]. There are studies suggesting no differences between subjective tolerability between medication with or without preservatives [1]. The principal actives alone have specific potential adverse effects on the cornea and ocular surface. Prostaglandin analogs are associated with meibomian gland dysfunction; beta-blockers reduce basal tear turnover rate by acting on receptors in the lacrimal gland; and alpha-adrenergic agonists have a higher incidence of ocular allergy [2]. Thus, even preservative-free glaucoma medication has deleterious effects on the ocular surface. In our sample, there was no statistically significant correlation between the principle actives and the SPEED score; however, alpha-adrenergic agonists were the ones with an almost significant relationship (p-value of 0.066) which may be related to the intolerance reported with topical medications such as brimonidine that may also predispose patients to ocular allergy from additional topical antiglaucoma drops [1]. In other studies, brimonidine has also been reported as the one that leads to more DED symptoms [6].

Overall, we observed a SPEED score relatively low with an average of 2.75, which means that in our sample, most patients had a mild DED and 25% of the patients did not complain about any symptom (SPEED score of 0). Despite the fact that a high percentage of patients were on eye drops with preservatives, we believe this did not lead to a higher rate of DED symptoms mainly because of two reasons. First, patients could have devalued these symptoms and underreported them. Second, some patients might already have been on treatment with artificial tears simultaneously to the glaucoma medication which could have reduced their symptoms. On the other hand, it is known that continuous exposure to preservatives decreases neuronal corneal nerve responses and density [15], which could underestimate the sensation of DED by the patient. This was a subjective-based evaluation, and no objective evaluation was performed in order to determine clinical signs of ocular surface disruption. Many patients were on glaucoma drops for a long time (more than five years) which may also have led to some habituation to the adverse effects of the eyedrops and subsequently less valorization of them.

Our study had some limitations. First, as stated before, the fact that no objective evaluation was performed could have underestimated the percentage of DED present in our sample. Second, no comparison group was inquired. Inclusion of a comparison group of patients without glaucoma would be useful since some studies have shown that patients without glaucoma might complain more about DED symptoms than patients with glaucoma since this last group tends to be more concerned about their disease and neglect other ocular symptoms [7]. It would be interesting to evaluate if this was the case in our population either. Third, we could have taken more robust statistical conclusions with a larger sample size. Finally, we should also have inquired patients about the usage of artificial tears simultaneously with IOP-lowering drops since they sometimes initiate its usage without our prescription.

## Conclusions

The results of our investigation were surprisingly positive in what concerns the presence of DED symptoms in our glaucoma sample, with a low SPEED score and only 8% of patients with moderate and another 8% with severe symptoms. This might be the result of the healthcare provider’s sensibilization to this issue and consequent early diagnosis of DED, early prescription of artificial tears and change from preservative to preservative-free medication on early signs of DED or from one class of medication to another.

It is very important to be aware of the potential development of ocular surface disease in patients with glaucoma, from the ones with no medication at all to the ones on topical medication, and have this aspect routinely observed during their appointments, to avoid a negative impact in their quality of life, avoid the possible discontinuation of glaucoma medication and improve their compliance with the treatment and also the efficacy of the medications. All of this will improve the prognosis of the disease and ultimately help preserve sight.
